# Nitrous oxide emissions from soils: how well do we understand the processes and their controls?

**DOI:** 10.1098/rstb.2013.0122

**Published:** 2013-07-05

**Authors:** Klaus Butterbach-Bahl, Elizabeth M. Baggs, Michael Dannenmann, Ralf Kiese, Sophie Zechmeister-Boltenstern

**Affiliations:** 1 Karlsruhe Institute of Technology, Institute for Meteorology and Climate Research, Atmospheric Environmental Research (IMK-IFU), Kreuzeckbahnstrasse 19, Garmisch-Partenkirchen 82467, Germany; 2 International Livestock Research Institute, Old Naivasha Road, Nairobi 00100, Kenya; 3 Institute of Biological and Environmental Sciences, University of Aberdeen, Zoology Building, Tillydrone Avenue, Aberdeen AB24 2TZ, UK; 4 Institute of Forest Botany and Tree Physiology, Chair of Tree Physiology, University of Freiburg, Georges-Koehler-Allee 53/54, Freiburg 79110, Germany; 5 Department of Forest and Soil Sciences, Institute of Soil Research, University of Natural Resources and Life Sciences Vienna, Peter Jordan Strasse 82, Vienna 1190, Austria

**Keywords:** N_2_O, processes, environmental controls, modelling

## Abstract

Although it is well established that soils are the dominating source for atmospheric nitrous oxide (N_2_O), we are still struggling to fully understand the complexity of the underlying microbial production and consumption processes and the links to biotic (e.g. inter- and intraspecies competition, food webs, plant–microbe interaction) and abiotic (e.g. soil climate, physics and chemistry) factors. Recent work shows that a better understanding of the composition and diversity of the microbial community across a variety of soils in different climates and under different land use, as well as plant–microbe interactions in the rhizosphere, may provide a key to better understand the variability of N_2_O fluxes at the soil–atmosphere interface. Moreover, recent insights into the regulation of the reduction of N_2_O to dinitrogen (N_2_) have increased our understanding of N_2_O exchange. This improved process understanding, building on the increased use of isotope tracing techniques and metagenomics, needs to go along with improvements in measurement techniques for N_2_O (and N_2_) emission in order to obtain robust field and laboratory datasets for different ecosystem types. Advances in both fields are currently used to improve process descriptions in biogeochemical models, which may eventually be used not only to test our current process understanding from the microsite to the field level, but also used as tools for up-scaling emissions to landscapes and regions and to explore feedbacks of soil N_2_O emissions to changes in environmental conditions, land management and land use.

## 1. Introduction

Nitrous oxide (N_2_O) is a long-lived trace gas in the atmosphere, with an average mixing ratio of 322.5 ppbv in the year 2009. Atmospheric N_2_O concentrations have increased by 19 per cent since pre-industrial times, with an average increase of 0.77 ppbv yr^−1^ for the period 2000–2009 [1]. There are mainly two reasons why the so-called laughing gas has been attracting a considerable interest of scientists. First, it is a potent greenhouse gas (GHG), with a 100-year global warming potential 298 times that of carbon dioxide (CO_2_; molecule for molecule) contributing 6.24 per cent to the overall global radiative forcing [1,2]. Second, it is the single most important depleting substance of stratospheric ozone [3]. The dominant sources of N_2_O are closely related to microbial production processes in soils, sediments and water bodies. Agricultural emissions owing to N fertilizer use and manure management (4.3–5.8 Tg N_2_O–N yr^−1^) and emissions from natural soils (6–7 Tg N_2_O–N yr^−1^) represent 56–70% of all global N_2_O sources [4].

**Figure 1.**
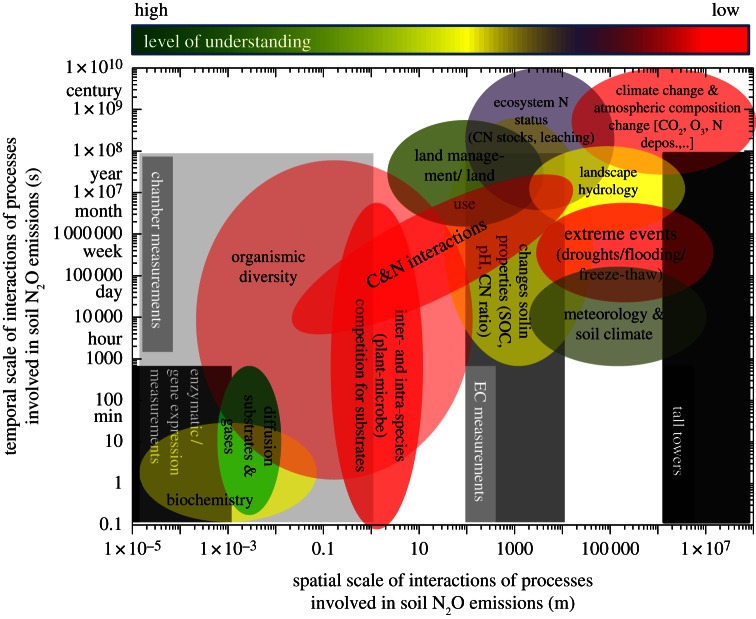
Drivers and processes of soil N_2_O emissions across temporal and spatial scales. Different colours indicate the level of understanding. Underlying grey boxes show different measuring techniques (enzymatic, chamber, eddy covariance (EC)/micrometeorological measurements) commonly used for identifying N_2_O production and consumption processes and soil surface fluxes.

Field measurements of N_2_O exchange between soils and the atmosphere across a wide variety of terrestrial ecosystems as well as laboratory incubation studies under controlled conditions—both with soils and with pure cultures of micro-organisms—provide an extensive set of measured emission fluxes. These measurements provide empirical estimates of emission over a range of scales spatially and temporally (figure 1).

However, up-scaling N_2_O budgets to national and regional scales remain an unresolved challenge with current national estimates still highly uncertain. This is mainly due to the very dynamic and variable character of N_2_O soil losses caused by a multitude of interacting controls [5]. As a result, soil N_2_O emissions are characterized by ‘hot spots’ and ‘hot moments’, i.e. by an enormous spatio-temporal variability [6–8]. Because the availability of reactive nitrogen (N_r_: here defined as organic bound N and inorganic N compounds except N_2_) is the major driver of N_2_O soil emissions, fertilizer use is a key factor controlling soil N_2_O fluxes [4,9]. However, elevated N_2_O soil fluxes are not only restricted to sites were N fertilizers are applied (the so-called direct emissions), but owing to volatilization, leaching and erosion processes, N_r_ is cascading from application sites to downwind and downstream ecosystems. This might result in natural ecosystem N enrichments, thereby creating new hot spots of N_2_O emissions (i.e. indirect emissions [10,11]). For a better understanding of N_2_O soil emissions, it is, on the one hand, necessary to understand nitrogen cycling from ecosystem to regional and global scales and on the other hand, to improve our understanding of key processes involved in N_2_O formation, consumption and emission. The challenge is to integrate the two.

Here, we summarize the current understanding of processes involved in N_2_O emissions, outlining advances and remaining challenges to characterize and quantify relevant soil processes and soil surface fluxes of N_2_O and describe the state of development of models used to simulate N_2_O soil fluxes from site to regional scale.

## 2. Production and consumption processes of nitrous oxide in soils

**Figure 2.**
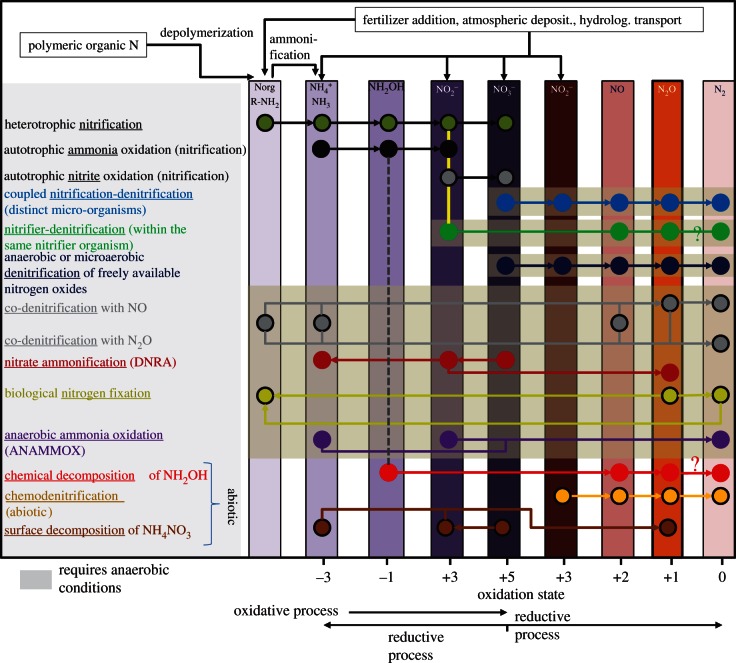
Biotic and abiotic processes of nitrous oxide (N_2_O). Processes potentially leading to N_2_O formation and consumption, involved N compounds, their reaction pathways as well as their oxidation states are shown. According to current knowledge, anaerobic ammonia oxidation does not contribute to N_2_O formation or consumption. By contrast, N_2_O may at least serve as a substrate for biological dinitrogen fixation. Processes predominantly requiring anaerobic (or micro-aerobic) conditions are underlined by grey illuminated segments. Norg/R-NH_2_, monomeric organically bound N forms; NH_4_^+^, ammonium; NH_3_, ammonia; NH_2_OH, hydroxylamine; NO_2_^−^, nitrite; NO_3_^−^, nitrate; NO, nitric oxide; N_2_O, nitrous oxide; N_2_, molecular dinitrogen. DNRA, Dissimilatory Nitrate Reduction to Ammonium.

Microbial nitrification and denitrification in managed and natural soils contribute approximately 70 per cent of global N_2_O emissions [4,12]. The description of microbial nitrification and denitrification as source of N_2_O is a simplification, because microbial metabolic pathways provide a wealth of processes that form or consume N_2_O. Furthermore, there are other abiotic processes producing N_2_O. To our current knowledge, the following processes contribute to N_2_O formation in soils (figure 2):

— chemical decomposition of hydroxylamine during autotrophic and heterotrophic nitrification,
— chemodenitrification of soil nitrite and abiotic decomposition of ammonium nitrate in the presence of light, humidity and reacting surfaces,
— nitrifier-denitrification within the same nitrifying micro-organism,
— coupled nitrification–denitrification by distinct micro-organisms (production of nitrate by nitrite oxidizers, which is immediately denitrified *in situ* by denitrifiers),
— denitrification conducted by organisms capable of using nitrogen oxides as alternative electron acceptors under O_2_-limiting environmental conditions,
— co-denitrification of organic N compounds with NO, and
— nitrate ammonification or dissimilatory nitrate reduction to ammonium.

For a detailed overview on the processes and references to relevant literature, see appendix, electronic supplementary material.

## 3. Techniques to characterize and quantify soil processes: tools, challenges and future perspectives

### (a) Inhibitors

The main inhibitor used to distinguish between nitrifier and denitrifier N_2_O production, which has been used in the past to quantify N_2_O + N_2_ production, is acetylene (C_2_H_2_). C_2_H_2_ gas at 10 Pa is applied to inhibit nitrification, and C_2_H_2_ at 10 kPa is applied to inhibit both nitrification and N_2_O reduction in denitrification [13]. In field situations, this C_2_H_2_ for inhibition of nitrification is sometimes produced from application of CaC_2_ granules [14]. The problems with this approach are now widely published. They include a systematic and irreproducible underestimation of denitrification owing to (i) a short supply of nitrifier-NO_3_^−^, (ii) decomposition of C_2_H_2_, (iii) oxidation of NO to NO_2_^−^ catalysed by high (more than 0.1%) concentration of C_2_H_2_ in the presence of oxygen and subsequently consumption of NO_2_^−^ by soil processes, (iv) utilization of C_2_H_2_ as a substrate for denitrification if C is limiting, (v) inhibition of nitrate ammonification (the extra pair of electrons that would have been used to reduce N_2_O to N_2_ can increase reduction of NO_3_^−^), and (vi) restricted diffusion of C_2_H_2_ in fine-textured or water-saturated soil [6,15,16]. Understanding of the regulation of the denitrifier N_2_O reductase has improved [17,18], so that earlier studies under strictly anaerobic conditions may be reanalysed taking into account the C stimulation.

There are a range of urease and nitrification inhibitors that have been used to lower emissions and nitrate leaching, including *N*-(*n*-butyl) thiophosphoric triamide, hydroquinone, dicyandiamide and 3,4-dimethylpyrazole phosphate [19–21]. Traditionally, these have been synthetic, but biological nitrification inhibition (BNI), such as mediated through plant exudates, is now attracting interest [22]. Inhibition can arise from competition between plants and microbes for available NH_4_^+^, but the exudation of nitrification suppressing compounds by plants (e.g. *Brachiaria humidicola* [23]) has recently been proposed as a mode of inhibition. Identified inhibitory compounds include free fatty acids, their methyl esters and a cyclic diterpene brachialoctone [24,25] which block both the ammonia monooxygenase and hydroxylamine oxydoreductase enzymes. The production of BNI compounds by crop species and their effectiveness in lowering N_2_O emission *in situ* has yet to be proved before BNI can be used in breeding programmes targeted towards environmentally sustainable food production.

### (b) Isotopes

Recent advances in stable isotope techniques have highlighted the contributions of various microbial groups to N_2_O emission from soil, and the influence of interactions between C and N cycle processes involved in the GHG production. These include both enrichment and natural abundance (^18^O, ^15^N) approaches [26]. N_2_O produced during nitrification is more depleted (more negative *δ*) in ^15^N and ^18^O relative to substrates than that produced during denitrification. This is partly due to N_2_O reduction in denitrification [27], which provides the opportunity for estimating the relative contributions of these two microbial processes. A natural abundance approach to source partitioning N_2_O production has been demonstrated to be of the greatest advantage in natural or unfertilized systems [28]. Natural abundance approaches have recently been used to identify the site preference (isotopomer) of ^15^N in N_2_O. This is the difference in *δ*^15^N between the central and outer N atoms in N_2_O, with different microbial processes and functional groups thought to exhibit distinct ^15^N-site preferences [29,30]. However, this approach is unable to distinguish denitrification by conventional denitrifiers from nitrate ammonification or ammonia oxidizer denitrification, so on its own is limited in the extent to which it will enable us to attribute N_2_O emission to different microbial sources.

Enrichment approaches have been used in fertilized systems, allowing the quantification of N_2_O produced during different processes. These have mostly focused on distinguishing between nitrification and denitrification following addition of ^15^N–NH_4_^+^ and/or ^15^N–NO_3_^−^ to soil [31,32]. Distinguishing between denitrification by conventionally defined denitrifiers and ammonia oxidizers remains problematic. A ^15^N/^18^O enrichment approach has recently been used by Wrage *et al*. [33], but there is still the risk of exchange of applied ^18^O in H_2_O with that of soil water and nitrate pools [34,35]. It may be possible for ammonia oxidizer denitrification (nitrifier denitrification) to be better constrained by coupling isotopic and molecular approaches (see below).

While these isotope approaches offer us the potential to determine the contribution of different microbial processes, they have not yet been applied to distinguish between all known microbial sources of N_2_O simultaneously. For example, the fractionation during nitrate ammonification has yet to be determined, and it may be that a combination of enrichment, natural abundance and isotopomer approaches coupled with molecular approaches may be required to estimate the contributions of all known N_2_O-genic processes.

### (c) Molecular techniques

It is only recently that molecular-based analyses of microbial diversity have been combined with measurements of N_2_O production and process rates. There have only been a few studies that offer a rigorous assessment of the microbial community coupled to a rigorous measurement of N_2_O production rates, or different microbial sources of N_2_O, but these provide conflicting results on the relationship between diversity and emissions. For example, Philippot *et al.* [36] demonstrate a significant correlation between the distribution of N_2_O-reducing bacteria and potential N_2_O emissions that appeared to be driven by soil pH, whereas in another study [37], no relationship between N_2_O : N_2_ ratio and denitrifier community size or composition after addition of C compounds to soil was found. Gene copy number analysis may provide a closer relationship with measured process, as a recent report showed significant relationships between *nirS*, *napA* and *narG* denitrification genes and the N_2_O/(N_2_O + N_2_) ratio from grassland soil [38]. Fewer studies have related ammonia oxidizer diversity or gene copy number to a quantification of ammonia oxidizer N_2_O production. Avrahami & Bohanann [39] report a significant relationship between ammonia oxidizer diversity and N_2_O emission rates and attribute spatial variation in N_2_O emissions to the composition of the ammonia-oxidizing community. However, there are other studies [40] that conclude that any change in ammonia oxidizer N_2_O production is the result of physiological responses rather than a change in the community composition. This highlights the need for further studies combining analysis of microbial ecology and quantification of N_2_O : N_2_ production and partitioning between the different microbial sources of N_2_O (see also appendix, the electronic supplementary material). A better insight into the regulation of these processes can be used to modify management practices to lower emissions.

### (d) Nitrification, denitrification, the N_2_ : N_2_O emission ratio and N_2_ : nitrification ratio at field scales

Our understanding of underlying processes, pathways and controls of N_2_O formation is still mainly based on studies with pure cultures of micro-organisms and soils under controlled conditions. However, a thorough understanding of N_2_O fluxes at various spatio-temporal scales requires an understanding of N cycling and loss rates of N_2_O during key microbial N transformation processes. Even though there is an increasing wealth of data on actual rates of nitrification and denitrification in soils, still little is known about N_2_O production and consumption as well as N_2_ emissions at field to landscape scales ([15]; figure 1).

This deficiency is mainly due to methodological problems of measuring N_2_ production by denitrification [41] and to disentangling N_2_O production processes at field scale [15]. It is very well established that the acetylene inhibition method creates systematic and irreproducible underestimation of N_2_ production by denitrification under aerobic incubation conditions [16,41,42,43]. However, most likely owing to its simplicity, the acetylene inhibition method is still used in studies and reported in literature. Besides the acetylene inhibition method, few methods remain that allow insights into N_2_ and N_2_O production by denitrification: mainly the gas-flow helium incubation method [44,45] (see appendix, the electronic supplementary material) or the determination of labelled N_2_ following the application of ^15^N-labelled substrates [46].

The electronic supplementary material, table S1, summarizes all available datasets where N_2_ emissions have been either measured by ^15^N-labelling approaches or with the gas-flow helium incubation method and which do provide estimates for annual or seasonal N_2_ as well as N_2_O emissions. Compared with the work by Schlesinger [47]—who also considered estimates of denitrification and N_2_ formation as obtained by the acetylene inhibition method—it is obvious from the electronic supplementary material, table S1, that for all soils from different ecosystems (forest, agricultural and wetland) N_2_O: (N_2_O + N_2_) ratios obtained are significantly lower if measurements with the acetylene inhibition method are ignored. For example, Schlesinger [47] estimated that the mean N_2_O yield of denitrification from soils under natural or recovering vegetation is approximately 49.2 per cent, whereas, in our analysis, this value is 20.7 per cent (see the electronic supplementary material, table S1). This significantly changes the estimate of the human impact on terrestrial denitrification. Schlesinger [47] calculated that the total rate of denitrification is at present 17 Tg N yr^−1^ greater than in pre-industrial times. Excluding data from acetylene inhibition methods and using the data provided in the electronic supplementary material the estimate changes to 46 Tg N yr^−1^ if the Schlesinger calculation approach is used. This new estimate of changes in terrestrial denitrification is much more in-line with estimates by other studies [48,49], showing that methodological problems and a lack of understanding at the process level is still hampering the assessment of the consequences of perturbation of N cycling at regional to global scales.

## 4. Environmental controls of nitrous oxide fluxes at various spatial and temporal scales

### (a) Moisture and temperature control of soil nitrous oxide emissions

Soil moisture is a major driver of N_2_O emissions as it regulates the oxygen availability to soil microbes. N_2_O emissions have their optimum in the range of 70–80% water-filled pore space (WFPS) depending on soil type [50]. At higher soil moisture, the major end product of denitrification is N_2_. After a screening of 51 soils across Europe, this concept was only partly supported. Most soils had their optimum of N_2_O emissions under wetter conditions than 80 per cent WFPS (see the electronic supplementary material, figure S1) [51]. Only a minority of soils (see the electronic supplementary material, figure S1) showed a decline in N_2_O emissions under wettest soil conditions, possibly owing to the rapid initialization of strictly anaerobic conditions, resulting in the formation of N_2_ rather than N_2_O. Seemingly, upland soils rarely reach moisture conditions that lie beyond the optimum for N_2_O emission.

Although soil moisture has a predominant effect on N_2_O emission, it was found that denitrification is extremely sensitive to rising temperatures. The Q_10_ of denitrification, i.e. the stimulation of denitrification following an increase in temperature by 10°C, exceeds the Q_10_ of soil CO_2_ emissions [52,53]. This fact can be attributed to a tight coupling between the microbial C and N cycle. Hence, N_2_O emissions are not only directly affected by temperature effects on enzymatic processes involved in N_2_O production. Furthermore, temperature-induced increases in soil respiration lead to a depletion of soil oxygen concentrations and to increases in soil anaerobiosis, with the latter being a precursor and a major driver. Also, the succession of several temperature-sensitive microbial processes within the nitrogen cycle, which cascade reactive N compounds through its different oxidation states (N-mineralization, nitrification; figure 2) providing the substrate for denitrification, leads to a multiplying effect of temperature increase on N_2_O fluxes from soil. In terms of global environmental change, this means that a positive feedback effect of warming on soil GHG emissions can be expected to be greater for N_2_O than for CO_2_. However, substrate and moisture limitations of microbial N cycling processes under climate-change conditions may dampen the stimulating effect of temperature [5]. Nevertheless, an implementation of these findings into global climate-change models may considerably alter predictions of future atmospheric composition and expected severity of climate change.

The impact of global change drivers such as temperature and moisture on ecosystem processes is well studied when acting in isolation or with, at most, one interacting variable [54]. While it can be argued that we understand how both drivers interact mechanistically, we fail to predict how emissions may change if a third or fourth driver comes into play (such as enhanced CO_2_, ozone or nitrogen). This is due to the nonlinearity of involved processes and synergistic or antagonistic rather than simple additive effects of combined drivers, so that an understanding of the underlying mechanisms becomes much more difficult [55]. There might be a general trend for the magnitude of the responses to decline with higher-order interactions, longer time periods and larger spatial scales [54]. However, while effects of dampening with scale and treatment complexity might be part of intrinsic system behaviour, threshold effects and tipping points which are so far not understood have to be taken into consideration when predicting global change effects.

Moreover, seasonal or spatial dynamics of soil moisture or temperature can affect N_2_O emission rates. Temporary waterlogging, seasonal passing from drought to rewetting (similar to the ‘Birch effect’ for soil respiration [56,57]) as well as transient zones between upland and wetland soils can constitute the so-called hot moments and hot spots for N_2_O emissions as they present ideal conditions for the transition from microbial oxygen to NO_3_ respiration [7]. Nitrous oxide reaction to changes in temperature will not always be the same depending on the state (e.g. substrate availability) of the soil system, which may result in hysteresis curves as also observed for soil CO_2_ respiration [58]: N_2_O release during rising temperatures can follow a different curve from falling temperatures owing to faster depletion of substrates (carbon compounds as well as nitrate). This is a phenomenon that needs to be better understood and accounted for in modelling.

Temperatures around 0°C are of special interest as many soil microbes are still active and freeze/thaw processes lead to pulses of N_2_O emissions with significant or dominant contributions to the annual N_2_O budget [7,8]. This may be driven by release of stored C during the thaw. It is these transition effects that still hold many secrets in the understanding of environmental controls of N_2_O release.

Often changes in soil moisture and soil temperature can explain up to 95 per cent of the temporal variations of field N_2_O emissions [59] constituting the main drivers of denitrification. The remaining unexplained gas fluxes are related to proximal drivers of oxygen supply, for example, substrate concentration, available energy and distal drivers of plant nitrate uptake, for example, litter/soil organic matter quality, root/microbial respiration, soil texture, predation, pH and pollution by heavy metals or organic chemicals [60].

### (b) How important is microbial diversity for soil nitrous oxide emissions?

The denitrifiers are a phylogenetic heterogeneous group of microbes. Mostly known are bacterial strains from the phyla Firmicutes, Actinomycetes, Bacteroides, Aquifaceae and α-, β-, γ- and *ɛ*-Proteobacteria [61,62]. They are also physiologically heterogeneous comprising nitrifiers, N_2_-fixers (symbiotic as well as non-symbiotic), thiosulfate oxidizers, methylotrophs, aerobic and anaerobic taxa, heterotrophs and autotrophs and even photosynthetic bacteria and extremophiles [63]. As highlighted earlier, denitrification can be classified as a microbiologically ‘broad process’ which can be conducted by a wide array of microbes in contrast to the comparatively ‘narrow process’ of autotrophic nitrification. Denitrifying bacterial communities tracked, for example, by their *nirK* genes encoding the nitrite reductase are therefore more diverse than their nitrifying counterparts detected by the ammonium monooxygenase-encoding (*amoA*) genes [64].

Although most knowledge on the denitrification process relates to bacterial denitrification, 20 years ago, some fungi [65,66] had already been reported to produce N_2_O. For example, N_2_O formation was observed in *Trichoderma harzianum* at anaerobic incubation with NO_2_ as N source. Fungal denitrification physiologically acts as anaerobic (NO_3_) respiration. *Fusarium oxysporum* and *Aspergillus nidulans* use dissimilatory ammonia fermentation—reducing nitrate to ammonium and simultaneously oxidizing ethanol to acetate. Ammonia fermentation and denitrification are alternatively expressed depending on the extent of the oxygen supply. Several fungal species belonging to the Ascomycetes and Deuteromycetes can form N_2_O from nitrite, and some can reduce nitrate under anaerobic as well as micro-aerobic conditions.

Although these fungi frequently occur in soils, and are especially abundant in the litter layers of forests, there is very little experimental evidence on their overall contribution to N_2_O emissions. Field studies about the role of fungi in denitrification are rare and methodologically hazardous as biocides are used to distinguish fungal from bacterial activity. The applied inhibitors, usually cycloheximide and streptomycin, can have multiple side-effects on the nitrogen cycle. It has been suggested that consideration of the position of ^15^N within the N_2_O molecule could help distinguish bacterial and fungal denitrification [67]. So, although there are reports on the importance of fungi for N_2_O formation in temperate, semiarid grasslands, woodland and tropical arable peat [68–70], new technologies are required to clarify the fundamental question—‘what *really* is the ecological role of fungi in denitrification?’

Within the domain of archaea the nitrite reductase encoding the *nirK* gene has been identified among extreme halophiles [71], however, N_2_O emission by soil archaea has so far never been proved. By now, it is known that archaea are numerous and widely distributed in soils around the world [72] and they even dominate microbial communities in boreal areas (C. Schleper 2011, personal communication). A reason for the lack of knowledge on archaeal physiology is the fact that they are extremely difficult to culture. It was reported that marine archaeal ammonia oxidizers may release N_2_O. These measurements were based on two archaeal enrichment (not pure) cultures [73]. Comparing ^15^N and ^18^O signatures, the authors suggested that ammonia-oxidizing archaea may be largely responsible for the global oceanic N_2_O source. *Nitrososphaera viennensis* is the first ammonia-oxidizing archaeon from soil to be grown in pure culture and its carbon and nitrogen metabolism were recently characterized [74]. It remains to be shown whether soil-inhabiting archaea produce N_2_O, which might have large implications for our current understanding of N_2_O soil emissions.

Although the ability to denitrify, as determined by the analysis of genes involved in denitrification, is widespread, the process is facultative and induced only under particular conditions [75]. One of the most outstanding questions microbial ecologists face is whether microbial communities that differ in composition also differ in function [75]. Although differences of denitrifier abundance may relate to varying denitrification enzyme activities [76,77], there is rare evidence for a correlation between denitrifier abundance and soil N_2_O emission. This suggests that the relative activity of the enzymes involved in denitrification may sometimes be affected by denitrifier composition but in other cases environmental factors may be the dominant determinants of activity.

In contrast to denitrification activity, bacterial denitrifier communities as represented by the total gene pool seem to be highly resistant to changes. Major modifications of the community structure were observed in long-term experiments by which the soil's physical and chemical parameters were also modified [78], whereas many laboratory experiments resulted in minor modifications [13]. The studies conducted so far suggest a redundancy of bacterial functional genes involved in denitrification. So even if community changes are observed, we do not know if a change in the diversity or composition of the denitrifier community will change denitrification activity or N_2_O fluxes [61].

The above-mentioned caveats might be overcome by further methodological developments. Most studies on denitrifying communities use methods to fingerprint the potentially involved microbes by using terminal restriction fragment length polymorphism analysis or denaturating gradient gel electrophoresis of PCR-amplified functional genes such as *nirS*, *nirK* and (less frequently) *nosZ*, owing to the availability of suitable primers. However, although the enzymes encoded by these genes are involved in the denitrification process, they do not release N_2_O, but either its precursor NO or its successor N_2_, which might be one of the reasons for missing relationships between results of molecular studies and *in situ* N_2_O fluxes (see the electronic supplementary material, figure S2). Only minuscule amounts of soil are generally used for DNA extraction thereby making it difficult to capture soil heterogeneity. It is important to be able to up-scale the results from 1 g of soil to the field or landscape; therefore, sampling strategies for DNA analysis have to be optimized in order to be representative at the landscape scale.

### (c) Ecosystem nitrogen saturation and nitrous oxide fluxes

The impact of increasing N deposition on natural ecosystems and their GHG emissions is still poorly understood [15]. Nitrogen saturation as reviewed by Aber *et al*. [79] may be defined as the availability of ammonium and nitrate in excess of total combined plant and microbial nutritional demand. The process leading to nitrogen saturation does not proceed linearly, but in different stages of which the last stage is postulated to be characterized by increased losses of N to the atmosphere (NO, N_2_O, N_2_) and the hydrosphere (NO_3_). It depends on the vegetation, the soil type, bedrock and climate how much nitrogen can be retained by the system before it reaches N saturation. For example, a N-limited boreal forest may take longer to become nitrogen saturated than a forest that is well supplied with nitrogen.

Ambient N inputs into natural forests vary from 2 to 60 kg ha^−1^ yr^−1^ in Europe [80]. Elevated N inputs into natural ecosystems could be expected to raise N_2_O emission rates. Indeed, it has been found that increasing NH_4_^+^ wet deposition led to increases in N_2_O emission at forest sites [81,82]. Similarly, increased N_2_O emissions were found on transect plots close to a poultry farm receiving elevated N deposition [83]. These dose–response relationships were observed at site scales characterized by homogeneous conditions. It is more difficult to detect significant relationships between N deposition and N_2_O emissions across forests at larger scales with higher heterogeneity. On a regional level (40 km distance), higher soil N_2_O and NO emissions occurred in a beech forest with higher N deposition [59]. Here, between 3.5 and 4.7 per cent of deposited N was re-emitted in the form of N_2_O. There was a strong correlation between N deposition and N emission over time, which shows that low N-input sites are especially responsive to increasing N inputs. As deciduous forests, and especially beech forests, are the forest types emitting most N_2_O, these relationships have to be considered carefully.

On a global level (for Europe, see the electronic supplementary material), a meta-analysis of ambient N_2_O emission reports from 23 studies revealed no clear dose–response effect for N deposition and N_2_O emission [84]. However, N fertilization (ranging from 10 to 562 kg N ha^−1^ yr^−1^) significantly increased N_2_O emission by an average 216 per cent across all ecosystems (agriculture aerobic/anaerobic, coniferous, deciduous, tropical forest, wetland, grassland, heathland). Furthermore, the meta-analysis revealed a higher N-induced emission factor of 1.43–1.90 across all terrestrial ecosystems compared with the factors calculated for agriculture, which was ranging from 1.0 to 1.2 [85,86]. For non-agricultural ecosystems (*n* = 42), Liu & Greaver's [84] study approximated an enhancement of N_2_O emissions of 0.0087 ± 0.0025 kg N_2_O–N ha^−1^ yr^−1^ per 1 kg N ha^−1^ yr^−1^ added to the ecosystem. Compared with other ecosystem types, tropical forests emitted more N_2_O under N enrichment (on average +739%) [84] which points towards unexpected strong feedbacks of soil N_2_O emissions to increasing atmospheric N deposition in the tropics, a currently observed phenomenon [87]. Among the five chemical forms of N fertilizer assessed [84], NO_3_^−^ showed the strongest stimulation (an average of +493%) of N_2_O emission. The mean response ratio from short-term studies was significantly higher than that of long-term studies.

It should be noted that N_2_O emissions are influenced by multiple interactions of soil, climate and vegetation, which may obscure the nitrogen effect, e.g. the N_2_O-to-N_2_ ratio may differ between sites and N saturation on sandy soils may promote NO_3_ leaching rather than N_2_O emission. These obscuring effects have to be dissected in order to better understand the true mechanisms behind the impacts of N input.

## 5. Shortcomings of available nitrous oxide flux measurement techniques from soil to landscape scales

Owing to the dependency of microbial N_2_O production and consumption processes on environmental controls such as substrate availability, redox potential and temperature, N_2_O fluxes from soils are notoriously variable across various temporal and spatial scales. However, understanding spatial variability of N_2_O fluxes is essential to better constrain the magnitude of soil–atmosphere exchange of N_2_O and to design statistically valid measurement programmes to determine flux rates from plot to regional levels.

To date, the most widely used measuring technique for quantifying soil N_2_O fluxes is the closed chamber technique. This is simple to use, inexpensive and allows us to study treatment effects as well as to carry out specific process studies. However, it also has severe shortcomings owing to effects on environmental conditions (e.g. temperature effects, soil compaction, plant damage, disturbance of diffusion gradients; [88,89]), limited coverage of soil surfaces (usually less than 1 m^2^) so that the spatial heterogeneity is often not sufficiently addressed, collar insertion in the soil and cutting of roots or with regard to the temporal coverage of measurements [90]. Owing to manpower constraints, the latter is often limited to weekly-to-monthly measurement intervals, so that estimates of the contribution of fluxes during peak emission periods, for example, following fertilizer application or during spring–thaw periods, are often associated with high uncertainties. Although the problem of the temporal coverage of flux measurements is increasingly addressed by using automated chamber systems, the problem of the spatial representativeness of chamber-based measurements cannot be easily solved. Spatial variability occurs not only in agricultural but also in natural systems [91,92] and is often driven by small-scale changes in soil properties (texture, soil organic carbon, gas diffusivity or water availability), plant cover or nutrient availability.

One upcoming new method for investigating spatial variability of trace gas fluxes is the use of the fast-box method [93]. Here, a chamber is linked to a fast and precisely operating N_2_O analyser (e.g. tunable diode laser, TDL). This allows a significant reduction in closure times, so that chamber positions can be changed in minutes, and spatial variability can be explored. By contrast, with standard gas chromatograph (GC) techniques, closure times of 30–60 min are common.

Following recent advances in measuring techniques, specifically owing to the commercial availability of laser instruments allowing high precision, accuracy and sensitivity as well as high temporal resolution (less than 1 Hz), the number of studies where micrometeorological methods (e.g. eddy covariance (EC) or gradient techniques) in conjunction with TDL or quantum cascade laser spectrometers are used to derive N_2_O fluxes for areas more than 0.5–1 ha is steadily increasing [94,95]. N_2_O flux measurements by micrometeorological methods allow small-scale variability of fluxes to be averaged and provide continuous observations of fluxes. The obtained flux estimate for a much larger area when compared with chamber techniques is fundamental for developing and testing up-scaling approaches. However, the technique is not appropriate in hilly terrain. Nevertheless, a combination of chamber and EC measurements provides both the landscape fluxes required for up-scaling and the fine spatial data needed to study processes.

To deepen the understanding of landscape-scale N_2_O fluxes, it will be necessary to further consider topographic effects on soil environmental conditions [96] and, thus, on microbial production and consumption processes involved in N_2_O emissions. Furthermore, an explicit approach is needed to deal with the effects of the dispersion of nitrogen downwind and downstream of its application area, i.e. to quantify not only direct N_2_O emissions owing to fertilizer application at a given site, but also indirect emissions from soils at landscape and catchment scales owing to the cascading of nitrogen [10,11]. A way forward to get a better understanding of the importance of indirect emissions and a quantification of the split of indirect versus direct N_2_O emissions at landscapes is the application of ^15^N isotopes in the scope of a medium-size catchment study (0.3–1 km^2^), with a catchment comprising different land-use and land-management types. This has so far not been done owing to costs for ^15^N fertilizers and the limited ability to measure specific N_2_O isotopes to the required precision. Nevertheless, having in mind the dynamic development of laser spectroscopy of N_2_O, which already allows a high accuracy of measurements of N_2_O isotopomers, a sufficient measuring precision is fast becoming feasible.

## 6. Modelling nitrous oxide emissions from terrestrial ecosystems

Modelling approaches are needed to estimate N_2_O emissions at various temporal and spatial scales, to assess different mitigation options and to understand and predict feedbacks of global changes (here climate, land-use and land-management changes). These can be simple empirical relationships as emission factor approaches or process-based biogeochemical modelling. Emission factor approaches such as the Intergovernmental Panel on Climate Change approach [97] are a valuable and, at national to continental scales, robust tool to estimate annual N_2_O emissions associated with agricultural practices and land-use change [98]. However, such approaches become inaccurate or fail at finer spatial or temporal scales. Moreover, available emission factor approaches do not account for all management practices that may be implemented to reduce N_2_O emissions from agriculture (different fertilizers types, intercropping, etc.) and are not able to describe the consequences of changing environmental conditions (e.g. prolonged drought periods) on N_2_O fluxes. This failure is a consequence of the highly complex interplay of numerous microbial processes at various spatial and temporal scales such as mineralization, nitrification, denitrification, immobilization, plant N uptake and plant litter production as well as physico-chemical processes such as volatilization, leaching and chemodenitrification (figures 1 and 2) that cannot be described by simplistic empirical emission factor approaches.

**Figure 3.**
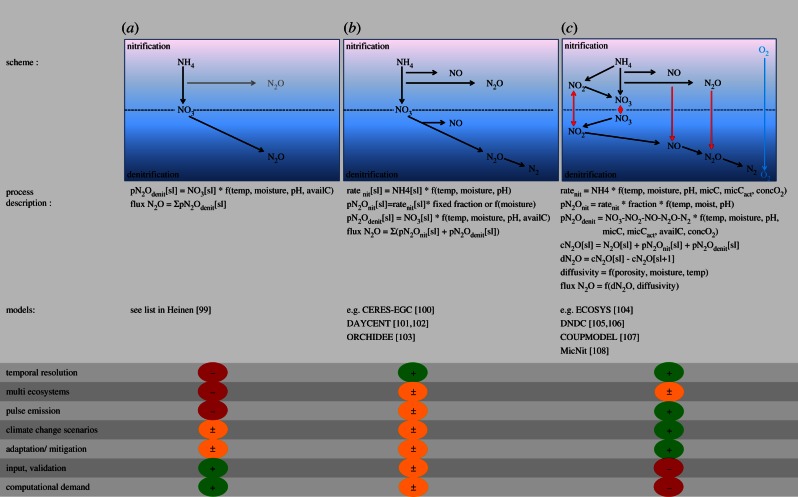
Schematic of process models used for simulation of N_2_O emission with different degrees of complexity: (*a*) simplified, (*b*) conceptual, (*c*) complex. Black arrows and components are accounted for in the models, grey arrows and components are optional, red arrows indicate exchange of components between anaerobic (denitrification) and aerobic (nitrification) micro-sites in the soil. Simplified process models use potential denitrification rates which are decreased by reduction factors related to soil environmental conditions for calculation of N_2_O emission. In addition, conceptual models also include N_2_O emission from nitrification mostly by use of fixed fractions. However, both simplified and conceptual models follow the theory that N_2_O production in the soil equals N_2_O flux at the soil–atmosphere interface. Complex process models calculate N turnover via nitrification and denitrification considering the dynamics of microbes. Nitrification and denitrification N turnover is weighted by calculation of anaerobic-aerobic volume fractions as function of soil oxygen concentrations. For this complex process models take into account diffusion processes which also determine the N_2_O flux at the soil–atmosphere interface, thus in contrast to simplified and conceptual models emission is not equal to production.

Within the past decades, a large number of process models have been developed for simulating soil N_2_O emissions applicable either only to one or to several specific ecosystem types (e.g. arable, grassland, forest; [99]). Models can be classified depending on their degree of complexity of descriptions of the main biogeochemical N turnover (mineralization, nitrification, denitrification) and trace gas production, consumption and emission processes into (i) simplified, (ii) conceptual and (iii) complex ecosystem models (figure 3).

Simple models follow the concept of calculating a potential denitrification rate which is subsequently modified to an actual denitrification rate by applying reduction factors that depend on actual environmental conditions such as soil temperature, moisture, pH and substrate availability (figure 3*a*). The reduction functions have to be parametrized independently for different model approaches and are mostly site or ecosystem specific. Moreover, they are semi-empirical, derived from field and laboratory experiments, thereby lumping together different driving factors for microbial processes (e.g. temperature and anaerobiosis). Therefore, these models may be used to reasonably predict the seasonal pattern of N trace gas emissions from soils for a given site, whereas their capability for higher time resolution (e.g. daily) and other sites is generally poor. The well-documented, high short-term dynamics of nitrogen transformation and associated N_2_O emission are driven by complex interactions between microbiological, plant and physico-chemical processes such as gas diffusion or solution–dissolution processes. Therefore, for a more realistic simulation of N_2_O emission patterns, such interactions need to be represented in the respective models in more detail [109].

One approach is the adaptation of the conceptual ‘hole in the pipe’ model ([110]; figure 3*b*). This concept describes emissions of N_2_O and NO from soils as a consequence of nitrogen transformations by denitrification and nitrification, with environmental conditions driving process-specific loss rates. Among environmental drivers, soil moisture is often regarded as the most important one. Soil water content in combination with soil physical properties (bulk density, texture)—the latter determining total porosity and pore size distribution—is so important because it controls the diffusion of oxygen into the soil. The availability of oxygen is of decisive importance not only for the oxidative process of nitrification, but also for the reductive process of denitrification where oxidized N compounds are serving as alternative electron acceptors. However, oxidative and reductive processes may occur simultaneously in different soil micro-aggregates [106,111], making it complex to numerically describe N_2_O production/consumption processes in soils.

The central role of soil oxygen status for controlling N turnover via nitrification and/or denitrification has been acknowledged and has led to a more explicit description of soil hydrology and soil gas transport mechanisms in complex ecosystem N cycling models ([106, 112–114]; figure 3*c*). The more detailed description of oxygen diffusion and consumption processes in soils allows the estimation of the oxygen concentration in a given soil layer and its use as a proxy to divide the soil into aerobic and anaerobic areas. This allows simultaneous simulation of nitrification and denitrification in a given soil layer [106]. Furthermore, the explicit consideration of oxygen diffusion as well as of concentrations of gaseous N compounds in the soil atmosphere is a prerequisite to simulate N_2_O and NO consumption processes. For example, NO or N_2_O produced by nitrification and being released to the soil atmosphere can in the next time step either be consumed by denitrification or diffuse to the next soil layer before gases are finally emitted to the atmosphere. Thus, most advanced models mimic the complex interplay of production, consumption and diffusion processes involved in soil N_2_O emissions. In recent years, continuous measurements with high temporal resolution revealed the importance of pulse emission events such as frost–thaw and re-wetting events for the annual N_2_O source strength of a given terrestrial ecosystem [7,115]. To simulate such events, more complex, diffusion-based models can be used to describe microbial biomass and activity dynamics by simulating the sequential biochemical reactions of nitrification and denitrification, for example, the stepwise activation of enzyme chains in dependence of substrate and oxygen availabilities [106,108,109].

The increasing complexity of models introduces additional uncertainties where model parameters cannot be clearly constrained. Therefore, to assure and improve the applicability of complex process models, parametric (as well as structural) uncertainties need to be quantified [116–119]. However, estimation of parameter optimization and uncertainty quantification for parameter-rich complex ecosystem models is still constrained by the high computational demand and the often insufficient structure of existing model codes ([120,121]; see also electronic supplementary material).

Increasing the model complexity is also required, because analysis of global change feedbacks on ecosystems and development of mitigation and adaptation strategies requires a multi-target view. The focus is not just N_2_O soil fluxes but also emissions and losses of other environmentally harmful Nr compounds (NO_3_^−^, NH_3_, NO*_x_*), ecosystem eutrophication and its effect on ecosystem biodiversity or changes in ecosystem C and N stocks. With regard to N cycle models, the view should be broadened, because the ecosystem view is often too narrow to represent a specific site. Ecosystem N inputs often depend on external inputs of reactive nitrogen by, for example, atmospheric deposition to a forest ecosystem or lateral water and nitrate influx in riparian areas. This takes the ‘simulation problem’ from plot or site (one-dimensional) to landscape scale (two-, three-dimensional) and results in a most challenging research topic, i.e. to describe nutrient fluxes and the various transport, emission and deposition pathways in a numerical model to finally mimic biosphere–hydrosphere–atmosphere exchange processes for a given landscape [11]. So far, all ecosystem models used for simulating N_2_O emissions are one-dimensional, thus, are neglecting topographical effects on soil hydrology, and in particular, the lateral hydrological transport of nutrients, for example, to riparian zones. Coupling of water and nutrient cycles for simulation of N transport and losses at catchment or landscape scale have so far mostly been based on one-directional exchange of data [122]. Alternatively, existing hydrological models have been supplemented with simple biogeochemical process descriptions to allow a more detailed simulation of nutrient turnover and associated losses [123–125]. Recent developments and awareness of model coupling software enable the linking of different models, even written in different programming languages, which allows for bi-directional exchange of states and parameters between the coupled models [126–128]. This approach was recently followed by the newly developed Nitroscape model framework which lumps together atmospheric, farm, agro-ecosystem and hydrological models and allows the simulation of spatial and temporal nitrogen dynamics at the landscape scale. First simulation results illustrated the effect of spatial interactions between landscape elements such as arable land and forests for N fluxes and losses to the environment, thereby highlighting the importance of indirect N_2_O emissions following N deposition and N leaching. Also, these authors [129] highlight the importance of landscapes because they represent both the scale at which land-management decisions are taken and the scale at which environmental impacts occur.

It is apparent that quantifying the biosphere–atmosphere exchange of nitrogen is extremely complex, both owing to the wide variety of nitrogen forms and microbial processes that need to be considered (figure 2) and to the challenging problem to overcome spatial and temporal variabilities. Analysing and understanding N fluxes at site but in particular at landscape scale is thus a major emerging challenge that requires a close cooperation of modelling and measuring research communities [11]. This cooperation may deliver more comprehensive datasets guiding further improvement and testing of complex site and landscape model systems that may be the only tool allowing sufficient integration and testing of our increased scientific knowledge [11].

## 7. Conclusions

In recent years, knowledge on processes and fluxes of N_r_ and N_2_O has advanced tremendously. New tools and techniques (e.g. isotopes, metagenomics) allowed the study and identification of processes and microbial communities involved in N_2_O production and consumption. Translation of this knowledge into models has begun, with models being increasingly used as powerful tools in global change studies. However, it is also obvious that our understanding of soil N cycling processes and the importance of microbial diversity, for example, with regard to the magnitude and spatio-temporal dynamics of soil N_2_O fluxes, is still limited. New approaches for up-scaling processes and fluxes from microbial scale to soil micro-sites, fields, entire landscapes and regions are still required, despite the recent progress. To overcome these shortcomings, there is an urgent need for interdisciplinary cooperation and knowledge transfer, because, for example, communities working on soil microbial processes and microbial diversity, biosphere–atmosphere exchange or modelling are still rather separated and wider perspectives such as C and N interactions or links of the N cycle with hydrology at landscape to global scales often attract little attention.
